# National Immunization Campaigns with Oral Polio Vaccine Reduce All-Cause Mortality: A Natural Experiment within Seven Randomized Trials

**DOI:** 10.3389/fpubh.2018.00013

**Published:** 2018-02-02

**Authors:** Andreas Andersen, Ane Baerent Fisker, Amabelia Rodrigues, Cesario Martins, Henrik Ravn, Najaaraq Lund, Sofie Biering-Sørensen, Christine Stabell Benn, Peter Aaby

**Affiliations:** ^1^Research Centre for Vitamins and Vaccines (CVIVA), Bandim Health Project, Statens Serum Institut, Copenhagen, Denmark; ^2^Bandim Health Project, Indepth Network, Bissau, Guinea-Bissau; ^3^Institute for Clinical Research, University of Southern Denmark, Odense University Hospital, Odense, Denmark

**Keywords:** campaigns, child mortality, MDG4, oral polio vaccine, non-specific effects of vaccines

## Abstract

**Background:**

A recent WHO review concluded that live BCG and measles vaccine (MV) may have beneficial non-specific effects (NSEs) reducing mortality from non-targeted diseases. NSEs of oral polio vaccine (OPV) were not examined. If OPV vaccination campaigns reduce the mortality rate, it would suggest beneficial NSEs.

**Setting:**

Between 2002 and 2014, Guinea-Bissau had 15 general OPV campaigns and other campaigns with OPV plus vitamin A supplementation (VAS), VAS-only, MV, and H1N1 vaccine. In this period, we conducted seven randomized controlled trials (RCTs) with mortality as main outcome.

**Methods:**

Within these RCTs, we assessed whether the mortality rate was lower after-campaign than before-campaign. We used Cox models with age as underlying time and further adjusted for low birth-weight, season and time trend in mortality. We calculated the adjusted mortality rate ratio (MRR) for after-campaign vs before-campaign.

**Results:**

The mortality rate was lower after OPV-only campaigns than before, the MRR being 0.81 (95% CI = 0.68–0.95). With each additional dose of campaign-OPV the mortality rate declined further (MRR = 0.87 (95% CI: 0.79–0.96) per dose) (test for trend, *p* = 0.005). No other type of campaign had similar beneficial effects. Depending on initial age and with follow-up to 3 years of age, the number needed to treat with campaign-OPV-only to save one life was between 68 and 230 children.

**Conclusion:**

Bissau had no case of polio infection so the results suggest that campaign-OPV has beneficial NSEs. Discontinuation of OPV-campaigns in low-income countries may affect general child mortality levels negatively.

## Key Observations

- Randomized trials and observational studies indicate that oral polio vaccine (OPV) may have beneficial non-specific effects for child survival. However, the numerous OPV-campaigns implemented in low-income countries to eradicate polio have not been assessed for impact on child mortality.
- Within seven randomized trials conducted in Guinea-Bissau, we compared the mortality rate before-campaign and after-campaign. In a combined adjusted analysis OPV-only-campaigns were associated with 19% (CI 5–32%) reduction in mortality rate.
- Each additional dose of campaign-OPV was associated with a decline of 13% (4–21%) in the mortality rate.
- Other campaigns did not have similar effects.

## Introduction

WHO recently reviewed the potential non-specific effects (NSEs) of Bacille Calmette–Guérin vaccine (BCG), diphtheria-tetanus-pertussis (DTP), and measles vaccine (MV) (1, 2). BCG and MV were associated with large reductions in overall mortality, in the range of halving mortality; these effects were not fully explained by prevention of tuberculosis and measles infection. Hence, the vaccines had “non-specific effects” (NSEs). A growing number of immunological studies supports that vaccines can generate heterologous non-specific protection by inducing cross-reactive T-cells or by training the innate immune system (3–6).

WHO did not review OPV. Investigations of NSEs are complex because it is usually not possible to randomize children to vaccines already recommended. Natural experiments are needed (7–9). Such an opportunity arises if the vaccine being examined is administered in campaigns in populations with demographic surveillance. It is then possible to assess whether the mortality rate changes after-campaign compared with before-campaign.

Low-income countries, such as Guinea-Bissau, have experienced numerous national campaigns during the last 15 years. In the same period, the Bandim Health Project (BHP) in Guinea-Bissau conducted several randomized controlled trials (RCTs). We used this set-up to analyze how OPV campaigns affected the mortality rate within these RCTs. If OPV campaigns reduce the mortality rate, it would be evidence of NSEs since there has been no polio infection in Bissau in this period. We also examined whether campaigns with vitamin A supplementation (VAS), MV, and H1N1 vaccine conducted during the same period changed the mortality rate.

## Materials and Methods

Bandim Health Project has maintained a health and demographic surveillance system (HDSS) in urban Guinea-Bissau since 1978; the BHP currently covers around 100,000 individuals. The HDSS involves monthly registration of pregnancies, births, routine vaccinations at the three health centers in the study area, three-monthly home visits to children less than 3 years of age to register growth, morbidity, vaccinations, survival, and risk factors for child survival. Furthermore, BHP monitors all deliveries at the national maternity ward in Bissau. The children are followed intensively to be able to register all vital events and describe the mortality pattern.

During the last decades, we have conducted seven RCTs of early vaccination (BCG, OPV, or MV) or VAS; the RCTs are described elsewhere (10–17) and are briefly summarized in the supplementary material.

### Campaigns

Between 2002 and 2014, numerous national campaigns, including 15 OPV campaigns, affected the children of eligible age groups enrolled in the seven RCTs. All campaigns are presented in detail in Table S1 in Supplementary Material.

Until 2005, the annual national OPV campaigns were organized with two doses of trivalent OPV given with an interval of 1 month; OPV was administered with VAS in the second round. Each dose of OPV was counted as a campaign. Nothing else was administered in these campaigns. From 2010, the pattern has changed with OPV often being distributed together with VAS and Mebendazole and often with a 6 months interval between campaigns; monovalent and bivalent OPV has also been used. OPV was provided to children between birth and 5 years of age, whereas VAS was only administered after 6 months of age and Mebendazole only after 12 months of age (Table S1 in Supplementary Material). In recent years, coverage has been well over 90% when we assessed participation (Table S1 in Supplementary Material).

Vitamin A supplementation was given in several of the OPV campaigns. However, there were also several campaigns in which VAS was given alone or with Mebendazole (Table S1 in Supplementary Material). These campaigns have been analyzed as VAS-only campaigns.

In the analysis, we distinguish between OPV-only, OPV-with-VAS, and VAS-only campaigns. Since VAS was not given to children less than 6 months of age, all OPV-campaigns were inherently OPV-only campaigns for children under 6 months of age even if VAS or Mebendazole were provided to older children during the same campaign.

The OPV campaigns were implemented by staff from the three health centers in the study area. They formed teams that went from house-to-house. In some campaigns, the teams were accompanied by HDSS field workers who had lists of all children generated from the HDSS databases. The field workers registered presence of the children and whether they received the interventions on that day or had already received them elsewhere. This system is costly and we have not been able to maintain it in all campaigns. Coverage has usually been very high (Table S1 in Supplementary Material).

There were three MV campaigns in 2006, 2009, and 2012. Few children were affected by these campaigns as most of the RCTs had follow-up to 12 months of age and MV was only given after 9 months of age in the 2009 and 2012 campaigns.

In October 2010, a national campaign with H1N1 influenza vaccine was implemented for children aged 6 months to 5 years.

### Analyses and Statistical Methods

We have previously examined whether OPV had beneficial NSEs reducing the child mortality rate by examining whether the results of RCTs of childhood interventions differed before and after the RCT-participants were exposed to campaign-OPV. If campaign-OPV had beneficial NSEs, the prediction would be that the mortality rate ratio (MRR) between the intervention and the control groups in the RCTs would be smaller after the OPV campaign. This analysis has been conducted for RCTs of early MV (18), BCG (15), and OPV-at-birth (OPV0) (16); within all three RCTs, we observed that the intervention effect was stronger before the OPV campaigns, supporting that OPV might have beneficial NSEs.

**Figure 1:**
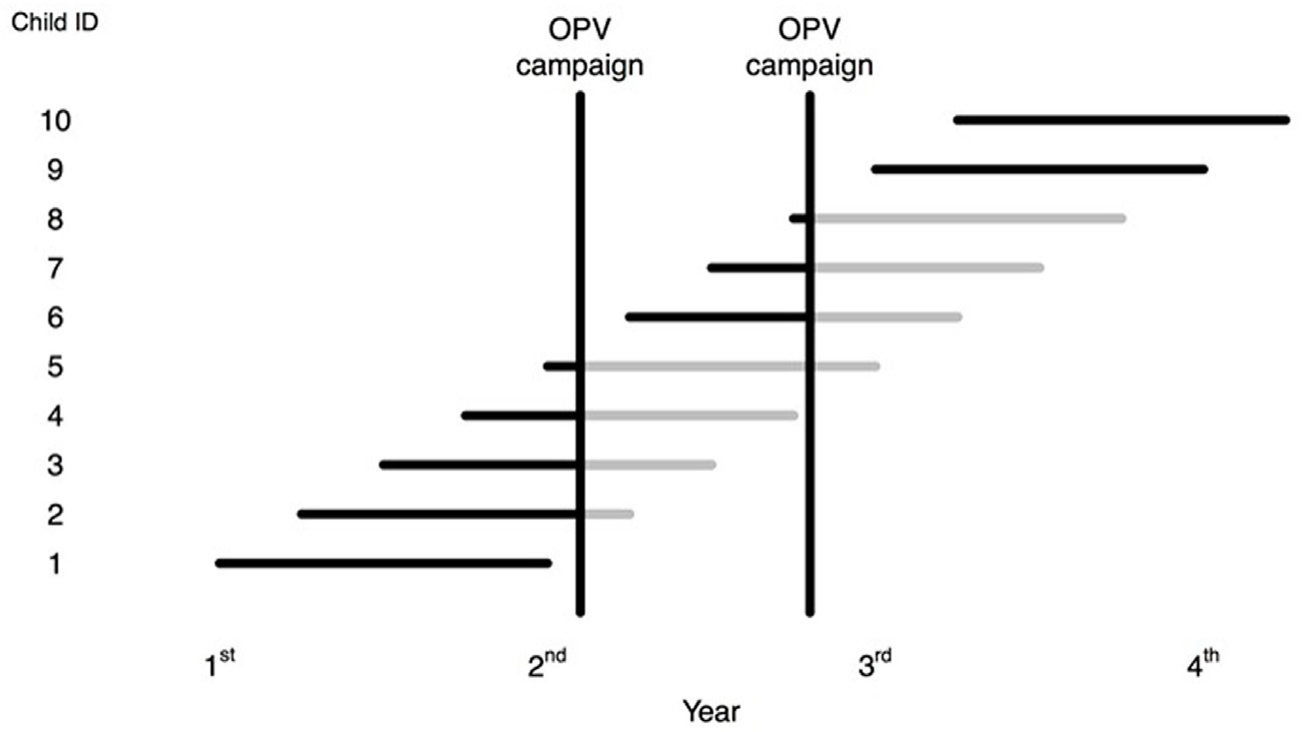
A hypothetically presentation of individuals in a trial: Time before oral polio vaccine (OPV) campaign in black and time after OPV campaign in gray.

To assess the potential NSEs of OPV in larger datasets, in the present paper, we estimate an intention-to-treat-effect or campaign-effectiveness effect comparing mortality after the campaign among eligible children (denoted after-campaign mortality) to mortality among children who lived in the population before the campaign, or who were born after the campaign (denoted before-campaign mortality). Hence, we are not comparing the mortality of the participating children and the few non-participating children. As illustrated in Figure 1, a child within any RCT could contribute observation time to both the before-campaign group and the after-campaign group. The randomness of birth dates and dates of enrollment in the trials as well of the randomness of the campaign dates determined how much of their follow-up time fell in the before-campaign period and how much in the after-campaign period.

We calculated the age-adjusted MRRs for after-OPV-only-campaign vs before-OPV-only-campaign in Cox-models with age as underlying time for each RCT and combined, overall and by sex. The before/after OPV-only-campaign variable was time-varying with the child changing status if it was eligible for the campaign. If a child experienced more OPV-only-campaigns, it continued to count in the after-OPV-only-campaign group. If a child experienced another type of campaign during follow-up, e.g., VAS-only-campaign, it counted with time-varying variables in both the after-OPV-only-campaign and the VAS-only-campaign groups from the respective dates of being eligible for these campaigns until the end of follow-up in the RCT.

The children in the two comparison groups (after-campaign vs. before-campaign) were all enrolled in RCTs with the same inclusion criteria. Hence, the children were already selected and were likely to constitute a rather homogeneous group, and hence there would be less risk of confounding from health and socio-economic background factors. The close follow-up in connection with the RCTs ensured good information on all children. As some RCTs were overlapping, we censored the children from one RCT once they entered another RCT. This implied that children in the neonatal VAS trials (RCTs I–II) and the LBW-BCG trials (RCT IV–V) were censored if they were enrolled at 4.5 months of age in the trials of early MV (RCT III).

Observational studies comparing the mortality of vaccinated and unvaccinated children may carry the risk of uncontrolled confounding because there are inherent differences between those who are vaccinated and those who are not. The design of the present analysis limited such confounding; the same kind of children were both controls and vaccinated, before the campaign they were all unvaccinated and after the campaign they were all assumed to have been vaccinated during the campaign. Hence, there was no question of healthy children being vaccinated and frail children not being vaccinated. All children were enrolled in RCTs that used similar inclusion criteria throughout the trials; hence, in an age-adjusted analysis, we compared similar children before and after the campaigns.

#### Multivariable Analysis

The main issue with splitting time before and after the campaigns is whether the overall trend in mortality or seasonal differences in mortality were allocated unequally to before-campaign and after-campaign. Due to the timing of the studies, the proportion of normal-birth-weight children and LBW children varied over the observation period from 2002 to 2014 and LBW children could be allocated unequally to before-campaign and after-campaign. Looking at each separate RCT, the campaign effect was difficult to disentangle from the effects of time and season on mortality.

We used the data from all seven RCTs to construct one large data set covering a period of 13 years in which we could control for year, season-at-risk (dry: 1 December–31 May; rainy: 1 June–30 November) and LBW (RCT IV–V). Subsequent tests (using Schoenfeld residuals) showed that these effects were age dependent. We, therefore, stratified by season and LBW, and modeled separate year-trends (year^⋆^agegroup) in the age groups 0–5, 6–11, and >12 months of age (using finer age intervals gave almost identical results).

The RCT-specific estimates of the OPV-only campaign effect were obtained from a model including the variables: OPV-only^⋆^study, study, OPV-with-VAS, VAS-only, MV, H1N1, year^⋆^agegroup, and stratified by season. The sex-specific estimates were obtained from a model, including OPV-only^⋆^study^⋆^sex, study^⋆^sex, OPV-with-VAS, VAS-only, MV, H1N1, year^⋆^agegroup, and stratified by season.

The RCT-specific estimates of OPV-with-VAS, VAS-only, MV, and H1N1 were obtained in similar models, including OPV-with-VAS^⋆^study, VAS-only^⋆^study, MV^⋆^study, and H1N1^⋆^study, respectively.

The combined overall effects of OPV-only, OPV-with-VAS, VAS-only, MV, and H1N1 were obtained from a single model, including the variables OPV-only, OPV-with-VAS, VAS-only, MV, H1N1, year^⋆^agegroup, and stratified by season and LBW. The sex-specific effects of OPV-only, OPV-with-VAS, VAS-only, MV, and H1N1 were obtained from a single model including OPV-only^⋆^sex, OPV-with-VAS^⋆^sex, VAS-only^⋆^sex, MV^⋆^sex, H1N1^⋆^sex, sex, year^⋆^agegroup, and stratified by season and LBW.

#### Goodness of Fit

We illustrated the fit of the multivariable model by plotting the observed mortality rates (deaths/100 person-years) in the three age groups: 0–5, 6–11, and >12 months vs the average of the predicted mortality rates in the same age groups (Figure S1 in Supplementary Material). We used a Poisson model equivalent to the multivariable Cox model to obtain predictions of the absolute mortality rates (instead of the relative mortality rates provided by the Cox model). In the Poisson model, we adjusted for age using the age groups 0, 1, 2, 4, 6, 8, 12, 18, and 24 months. LBW and season were included with interactions with the three age groups (opposed to stratifying by these in Cox). The Poisson model and the Cox model provided very similar results.

#### Sensitivity Analysis: Simulations

As a sensitivity analysis, we tested whether the estimated effect could be a spurious effect created by splitting time before and after campaigns. Fictive OPV campaign dates were created by simulation. Our seven RCTs covered a period of 13 years and there were seven years with OPV campaigns. In the simulations, the campaign dates were generated randomly such that there were OPV campaigns in 7 years in the 13-year follow-up period. In each simulation, it was decided randomly in which years the fictive campaigns occurred. Furthermore, the campaign date was assigned randomly within a given year. Time was split before and after these fictive campaign dates. The sensitivity analysis then estimated the OPV-campaign effect in a Cox-model with age as the underlying time. The simulation of fictive campaign dates was repeated 1,000 times and the average OPV-campaign effect was calculated.

#### Number Needed to Treat

Number needed to treat is calculated as [1/[*S*_-OPV_(*t*)^MRR^−S_-OPV_(*t*)] where *S*_OPV_(*t*) is the Kaplan–Meier estimate in the before-campaign-OPV group and MRR is the mortality rate ratio for campaign-OPV-only obtained from the multivariable analysis (19).

## Results

### OPV-Only Campaigns

After OPV campaigns, the mortality rate was reduced; the adjusted MRR for after-campaign vs before-campaign was 0.81 (95% CI = 0.68–0.95) (Tables 1–3). The effect of OPV-only campaigns was separately significant for boys [MRR = 0.74 (0.58–0.94)] but not for girls [MRR = 0.87 (0.70–1.07)] (Table 2). To assess whether the effect was stable over time we censored follow-up 6 months after the children was eligible to the first OPV-only campaign; in this analysis, the effect of OPV-only campaign was 0.78 (0.65–0.95).

**Table 1:** Mortality rates (per 100 person-years) and mortality rate ratios (MRR) for after-campaign vs before-campaign children enrolled in seven randomized controlled trials (RCTs), presented by campaign type.

Variable	Rate per 100 person-years (deaths/person-years)		MRR^#1^	MRR^#2^	MRR^#3^
	After-campaign	Before-campaign			
Campaign-OPV-only	2.04 (316/15,462)	5.05 (928/18,360)	0.81 (0.70–0.93)	0.83 (0.71–0.97)	0.81 (0.68–0.95)
Campaign-OPV + VAS	1.51 (117/7,761)	4.32 (1127/26,061)	0.99 (0.78–1.24)	0.95 (0.73–1.24)	1.10 (0.82–1.48)
Campaign-VAS-only	1.49 (176/11824)	4.86 (1068/21998)	0.92 (0.74–1.15)	1.06 (0.83–1.34)	1.04 (0.80–1.35)
Campaign-H1N1	3.06 (12/393)	3.69 (1232/33429)	1.14 (0.64–2.04)	1.65 (0.91–2.99)	1.86 (1.02–3.42)
Campaign-MV	2.03 (18/888)	3.72 (1226/32934)	1.10 (0.68–1.77)	1.16 (0.70–1.93)	1.24 (0.74–2.09)

*MRR^#1^: adjusting for age (underlying time)*.

*MRR^#2^: adjusting for age (underlying time), year^⋆^age group, strata (LBW, season)*.

*MRR^#3^: Full multivariable model: adjusting for age (underlying time), OPV, OPV + VAS, VAS, H1N1, MV, year^⋆^age group, strata (LBW, season)*.

*There were 1,244 deaths in the seven RCTs. Since “before-campaign” children were generally younger than the “after-campaign” children the MRR cannot be deduced directly from the mortality rates*.

**Table 2:** Mortality rate ratios (MRR) for trial participants after OPV-only campaigns compared with before OPV-only campaigns in seven randomized trials.

Trial Intervention	Recruitment period	Age group	MRR (OPV/noOPV)	MRR (OPV/noOPV)	MRR (OPV/noOPV)
			All	Males	Females
I. Neonatal VAS vs placebo NBW	2002–2004	0–11 months	0.62 (0.35–1.08)	0.61 (0.28–1.34)	0.64 (0.29–1.42)
II. Neonatal VAS vs placebo NBW 2-dose	2004–2007	0–11 months	0.92 (0.54–1.57)	0.94 (0.47–1.90)	0.89 (0.40–1.96)
III.MV at 4.5 + 9 months vs MV at 9 months	2003–2007	4.5–36 months	0.87 (0.62–1.23)	0.78 (0.50–1.21)	0.98 (0.64–1.52)
IV.BCG-at-birth vs delayed BCG to LBW children	2004–2008	0–11 months	0.65 (0.39–1.08)	0.45 (0.20–1.05)	0.82 (0.44–1.52)
V.BCG-at-birth vs delayed BCG to LBW children	2008–2013	0–11 months	0.71 (0.54–0.93)	0.63 (0.40–0.99)	0.75 (0.54–1.04)
VI.OPV + BCG vs BCG-only	2008–2011	0–11 months	1.13 (0.78–1.62)	1.05 (0.64–1.71)	1.24 (0.75–2.04)
VII.VAS vs placebo with vaccines; 12 months follow-up	2007–2010	6–29 months	0.45 (0.13–1.59)	0.23 (0.03–1.78)	0.87 (0.18–4.15)

Combined result			0.81 (0.68–0.95)	0.74 (0.58–0.94)	0.87 (0.70–1.07)

*The OPV campaigns were conducted in 2002, 2004, 2005, 2010, 2011, 2012, and 2013 (Table S1 in Supplementary Material). The effect of OPV-only campaigns was 0.90 (0.71–1.14) in the dry season and 0.73 (0.58–0.92) in the rainy season*.

**Table 3:** Overall model of impact on the mortality rate of different campaigns with oral polio vaccine (OPV).

Variable	Campaign OPV^(1)^ MRR	*p*-value	Campaign OPV^(2)^ MRR	*p*-value	Campaign OPV^(3)^ MRR	*p*-value
Campaign-OPV	0.81 (0.68–0.95)	0.011	0.87 (0.79–0.96)	0.007	0.77 (0.56–1.08)^m^	0.13
					0.80 (0.57–1.12)^b^	0.20
					0.85 (0.71–1.02)^t^	0.09
Campaign-OPV + VAS^a^	1.10 (0.82–1.48)	0.53	1.06 (0.79–1.41)	0.70	1.08 (0.81–1.44)	0.61
Campaign-VAS^a^	1.04 (0.80–1.35)	0.76	1.05 (0.81–1.36)	0.72	1.03 (0.80–1.34)	0.81
Campaign-H1N1^a^	1.86 (1.02–3.42)	0.04	2.10 (1.13–3.92)	0.02	1.90 (0.98–3.70)	0.06
Campaign-MV^a^	1.24 (0.74–2.09)	0.42	1.26 (0.74–2.12)	0.39	1.21 (0.72–2.04)	0.48
Calendar year, 0–5 months^b^	0.95 (0.93–0.98)	0.001	0.95 (0.93–0.98)	<0.001	0.96 (0.93–0.98)	0.001
Calendar year, 6–11 months^b^	0.89 (0.85–0.94)	<0.001	0.89 (0.85–0.94)	<0.001	0.90 (0.85–0.95)	<0.001
Calendar year, >12 months^b^	0.83 (0.73–0.94)	0.004	0.83 (0.73–0.94)	0.004	0.84 (0.73–0.96)	0.01

*^a^after-campaign vs before campaign*.

*^b^Calendar year trend in the age-groups of 0–5, 6–11, and >12 months of age*.

*^(1)^After-campaign vs before campaign*.

*^(2)^Continuous dose-response for OPV*.

^(3)^After-campaign vs before campaign; OPV-type: monovalent^m^, bivalent^b^, trivalent^t^.^.^

Oral polio vaccine was given from birth whereas the other interventions were only administered after 6 months. We, therefore, conducted a separate analysis for 0- to 5-month-old children; in this age group, the adjusted MRR for after-campaign vs before-campaign was 0.87 (0.69–1.09) (data not shown). OPV-with-VAS campaigns was usually given 1 month after OPV-only campaigns and they did not further change mortality in the model controlling for other campaigns, the MRR associated with OPV + VAS campaigns being 1.10 (0.82–1.48) (Tables 1 and 3).

We have found that boosting enhances the beneficial NSEs of live vaccines (9, 12, 20) and we, therefore, tested whether repeated doses of OPV-only in campaigns enhanced the beneficial effect. In the combined analysis adjusted for other campaigns, season, calendar year, and low-birth-weight, each additional dose of campaign OPV reduced mortality by 13% [MRR = 0.87 (0.79–0.96)] (Table 3). This effect was significant for boys [MRR = 0.80 (0.70–0.91)] but not for girls [MRR = 0.94 (0.83–1.05)] (*p* = 0.07, test-of-homogeneity).

There were no significant differences in effect of monovalent, bivalent, or trivalent OPV strains (Table 3).

### Other Campaigns

There was no significant overall effect of campaigns with VAS-only [MRR = 1.04 (0.80–1.35)] (Tables 1 and 4). Nor was there any significant overall effect of campaign with MV [MRR = 1.24 (0.74–2.09)] (Tables 1 and 5).

**Table 4:** Mortality rate ratios (MRR) for trial participants after VAS-only campaigns compared with before VAS-only campaigns in seven randomized trials.

Trial Intervention	Recruitment period	Age group	MRR (VAS/noVAS)	MRR (VAS/noVAS)	MRR (VAS/noVAS)
			All	Males	Females
I. Neonatal VAS vs placebo NBW	2002–2004	0–11 months	2.88 (1.58–5.24)	3.54 (1.71–7.33)	2.23 (0.91–5.42)
II. Neonatal VAS vs placebo NBW 2-dose	2004–2007	0–11 months	1.38 (0.70–2.70)	1.58 (0.67–3.71)	1.18 (0.42–3.31)
III. MV at 4.5 + 9 months vs MV at 9 months	2003–2007	4.5–36 months	0.92 (0.63–1.35)	0.96 (0.60–1.54)	0.91 (0.57–1.43)
IV. BCG-at-birth vs delayed BCG to LBW children	2004–2008	0–11 months	0.49 (0.25–0.95)	0.52 (0.21–1.32)	0.46 (0.18–1.15)
V. BCG-at-birth vs delayed BCG to LBW children	2008–2013	0–11 months	0.76 (0.39–1.50)	0.33 (0.05–2.42)	0.91 (0.44–1.84)
VI. OPV + BCG vs BCG-only	2008–2011	0–11 months	1.73 (0.94–3.19)	1.19 (0.47–3.03)	2.36 (1.09–5.10)
VII. VAS vs placebo with vaccines; 12 months follow-up	2007–2010	6–17 months	0.85 (0.33–2.21)	1.06 (0.29–3.81)	0.63 (0.16–2.40)

Combined result			1.04 (0.80–1.35)	1.07 (0.78–1.46)	1.02 (0.75–1.38)

*The VAS-only campaigns were conducted in 2003, 2006, 2007, 2008, 2009, 2010, 2012, and 2014 (Table S1 in Supplementary Material). The effect of VAS campaigns was 1.12 (0.71–1.75) in the dry season and 1.00 (0.73–1.36) in the rainy season*.

**Table 5:** Mortality rate ratios (MRR) for trial participants after measles vaccine (MV) campaigns compared to before MV campaigns.

Trial Intervention	Recruitment period	Age group	MRR (MV/noMV)	Males	Females
II. Neonatal VAS vs placebo NBW 2-dose	2004–2007	0–11 months	0.63 (0.09–4.60)	0	1.37 (0.19–9.98)
IV. BCG-at-birth vs delayed BCG to LBW children	2004–2008	0–11 months	0.77 (0.24–2.46)	0	1.42 (0.44–4.56)
V. BCG-at-birth vs delayed BCG to LBW children	2008–2013	0–11 months	0	0	0
VI. OPV + BCG vs BCG-only	2008–2011	0–11 months	3.10 (0.95–10.1)	5.98 (1.81–19.7)	0
VII. VAS vs placebo with vaccines; 12 months follow-up	2007–2010	6–17 months	1.65 (0.77–3.54)	1.71 (0.66–4.44)	1.51 (0.45–5.03)

Combined result			1.24 (0.74–2.09)	1.34 (0.68–2.64)	1.14 (0.55–2.38)

The inactivated H1N1 vaccine campaign affected three RCTs (Table 6). The H1N1 campaign was associated with an increase in mortality [MRR = 1.86 (1.02–3.42)]; this effect was only found for girls. The effects of OPV-only campaigns and H1N1 differed significantly (*p* = 0.01, test-of-homogeneity).

**Table 6:** Mortality rate ratios (MRR) for trial participants above 6 months of age after the H1N1 campaign compared with before the H1N1 campaign in 2010.

Trial Intervention	Recruitment period	Age group	MRR (H1N1/noH1N1)	Males	Females
V. BCG-at-birth vs delayed BCG to LBW children	2008–2013	0–11 months	2.16 (0.94–4.99)	^#^	2.28 (0.98–5.27)
VI. OPV + BCG vs BCG-only	2008–2011	0–11 months	1.44 (0.52–3.96)	0.71 (0.10–5.13)	2.19 (0.68–7.08)
VII. VAS vs placebo with vaccines; 12 months follow-up	2007–2010	6–17 months	6.48 (1.42–29.6)	4.63 (0.58–36.9)	9.84 (1.20–81.0)

Combined result			1.86 (1.02–3.42)	0.95 (0.23–3.92)	2.32 (1.19–4.52)

*^#^Prior to the campaign, only females were included in the randomized controlled trial and an estimate could, therefore, not be made for males*.

### Simulations

We conducted 1,000 simulations with fictive random OPV campaign dates. The average simulated OPV campaign effect on the mortality rate was 1.00 (0.99–1.01). The median was 0.99 and the inter-quartile-range of the simulated OPV campaign effects was (0.91–1.09).

### Number Needed to Treat

Depending on initial age, it was necessary to give campaign OPV to between 68 and 230 children to save one life within 3 years of age (Table 7).

**Table 7:** Number needed to treat (NNT) to save one life in accordance with initial age and length of follow-up.

	Age at start of follow-up: t_0_				
Follow-up length: (t_0_,t)	t_0_ = 0 months	t_0_ = 3 months	t_0_ = 6 months	t_0_ = 9 months	t_0_ = 12 months

1 year from start	*R*_−_ = 0.0585	*R*_−_ = 0.0298	*R*_−_ = 0.0230	*R*_−_ = 0.0209	*R*_−_ = 0.0147
	RD = 0.0109	RD = 0.0056	RD = 0.0043	RD = 0.0039	RD = 0.0028
	NNT = 92	NNT = 179	NNT = 231	NNT = 254	NNT = 361

2 years from start	*R*_−_ = 0.0724	*R*_−_ = 0.0436	*R*_−_ = 0.0374	*R*_−_ = 0.0310	*R*_−_ = 0.0231
	RD = 0.0133	RD = 0.0081	RD = 0.0070	RD = 0.0058	RD = 0.0043
	NNT = 75	NNT = 123	NNT = 143	NNT = 172	NNT = 230

Until 3 years of age	*R*_−_ = 0.0803	*R*_−_ = 0.0495	*R*_−_ = 0.0392	*R*_−_ = 0.0310	*R*_−_ = 0.0231
	RD = 0.0147	RD = 0.0092	RD = 0.0073	RD = 0.0058	RD = 0.0043
	NNT = 68	NNT = 109	NNT = 136	NNT = 172	NNT = 230

*The risk of dying from t_0_ to t for children alive at age t_0_ in the −OPV group is R_−_ = 1 − S(t| − OPV, alive at t_0_). S(t| − OPV, alive at t_0_) is obtained by the Kaplan–Meier method censoring children if they received oral polio vaccine. The risk in the +OPV group is calculated as R_+_ = 1 − S(t|−OPV, alive at t_0_)^0.81^ where 0.81 is the OPV-campaign mortality rate ratio from Table 1. The risk-difference is RD = R_−_ R_+_ and numbers needed to treat is NNT = 1/RD*.

## Discussion

### Main Observations

Oral polio vaccine provided in campaigns may have beneficial NSEs since it reduced the mortality rate substantially even though there was no polio infection. Additional doses of OPV were associated with further reductions in mortality. The NNT to save one life was very low. Other campaigns did not appear to have an overall beneficial effect.

### Strengths and Weaknesses

The beneficial effect of OPV campaigns in any RCT could have been related to the seasonality of campaigns or a general time-trend with declining mortality. However, the estimated beneficial effect was unchanged when we used all seven RCT data sets to adjust for seasonality and temporal effects. The simulations indicated that it would be unlikely to obtain the observed OPV campaign effect by chance. The fact that the same beneficial effect was not found for other campaigns (VAS-only, MV, H1N1) or when campaign dates were chosen at random in our simulations also supports that the observed beneficial effect of OPV campaigns was not due to seasonality of campaigns or a time trend in mortality. The H1N1 vaccine campaign was associated with increased mortality, particularly for girls. This is in line with the observation that other non-live vaccines, including DTP vaccine (21), Hepatitis B Vaccine (22), inactivated polio vaccine (23), pentavalent vaccine (24), and RTS,S malaria vaccine (25), are associated with increased mortality, particularly for females.

When we censored the analysis after 6 months of follow-up, the effect estimate was similar to the main result suggesting that the effect of OPV-only campaigns may last more than 6 months. Mortality is high in first months of life and neonatal deaths will occur mainly in the before-campaign group; however, the Cox survival analysis was strictly age-adjusted so this would not affect the effect estimates.

The effect of OPV-only campaigns appeared to be slightly stronger for boys than for girls, though the difference was not statistically significant. Other studies of OPV have also suggested a stronger beneficial effect for boys (16, 26).

In all the OPV campaigns, the coverage was higher than 80%, and in recent years higher than 90%. Not all children received OPV and the estimate should, therefore, be seen as an estimate of the effectiveness of OPV campaigns rather than as an estimate of the “true” effect of OPV.

We have previously found beneficial NSEs of MV campaigns reducing mortality in both urban and rural areas of Guinea-Bissau (27, 28). In the present analysis, there was no effect of the MV campaigns. However, there was limited follow-up time after MV because campaign MV was usually given after 9 months and most RCTs ended follow-up at 12 months of age. Hence, follow-up was mainly between 9 and 12 months of age and this is the age group in which MV is given routinely to children. Therefore, the study design was not optimal to evaluate whether campaign MV has an overall effect on mortality.

### Consistency or Contradiction with Previous Observations on OPV

The last 15–20 years have seen a large number of national OPV campaigns being implemented in low-income countries; the effect on overall survival was not tested before these campaigns were introduced. Studies from the 1960s when OPV was developed, reported that OPV reduced mortality in Chile and Brazil (29, 30) and Soviet Union researchers claimed that vaccination with non-pathogenic enterovirus, including OPV had general beneficial effects on health, including less respiratory infections (31).

When the first OPV campaigns were implemented in Guinea-Bissau in 1998, we examined the effect on child survival of having participated or not participated in the campaign (8). OPV was associated with significant benefits for the youngest children, reducing mortality and the risk of hospital admission. We have also found in a natural experiment, when DTP was missing, that the case-fatality at the pediatric ward was much lower for children who had received OPV-only and not OPV + DTP as currently recommended (9).

Recently, we conducted two RCTs of OPV0 in Bissau and found 32% lower infant mortality for children randomized to OPV0 who had not yet received campaign-OPV (16, 32).

One observational study has suggested that routine OPV0 compared with NoOPV0 might have a negative effect for boys but not for girls (33). However, the boys who had “benefited” from not having received OPV0 had been more likely to receive campaign OPV than the boys receiving OPV0 and this is likely to have been the real cause of their lower mortality (26). In the subsequent RCT to measure the effect, OPV0 was significantly beneficial, particularly for boys (16).

Studies from high-income countries have also suggested that OPV may have beneficial effects (34, 35). In a nation-wide study in Denmark, OPV given at 2 years of age (until 2001) was associated with a 15% (5–23%) reduction in hospital admissions compared with children who had DTaP-Hib-IPV as their most recent vaccination (35). Additional doses of OPV at 3 and 4 years of age were also associated with significant reductions in admissions (35). Hence, the effects of OPV observed in Guinea-Bissau may represent a true beneficial NSE.

### Interpretation and Immunological Mechanisms

The studies from both low-income and high-income countries suggest that OPV may reduce susceptibility to unrelated infections. In high-mortality settings such as Guinea-Bissau, essentially all deaths are due to infections. Hence, the observed association between OPV and lower general mortality likely reflects a reduced susceptibility to infections. In the RCT of OPV0, the children may have grown better if they received OPV at birth, which could also reflect lower risk of infection (16). In Finland, OPV was associated with reduced risk of otitis media (34); in Denmark, OPV was associated with reduced risk of lower respiratory infections (35). These data suggest that OPV, as MMR (36), may particularly affect the susceptibility to upper and lower respiratory infections. The studies of the effect of OPV-campaigns on the results in RCTs of childhood interventions also supported that campaign-OPV reduced the mortality rate and the difference between the interventions groups in the RCTs (15, 16, 18).

How OPV may affect susceptibility to infections in general has not been studied. Other live vaccines have been found to induce immune training effects that reduce susceptibility to unrelated infections (1, 13, 14). For instance, BCG reprograms monocytes through epigenetic changes to a more pro-inflammatory response; in animal models, this translates into reduced mortality from challenge to unrelated infections (3). Similar studies need to be conducted for OPV. Furthermore, heterologous T-cell immunity may induce cross-reactive T-cells, which have been shown to be an important determinant in subsequent unrelated infections (37, 38). OPV may presumably also change the microbiome (39).

There has been little research in why NSEs often differ by sex. Several studies have found a stronger beneficial effect of OPV for males than for females (16, 26). Other live vaccines, such as MV (1, 13, 27) may have a stronger beneficial effect for females. On the other hand, non-live vaccines appear consistently to have a stronger negative effect for females than for males (21–25). Though mechanistic explanations have not been detected, it may be important that females apparently have a stronger Th2 immune profile than males (40) and non-live vaccines induce a Th2-weighted response, which has been found to be deleterious upon rechallenge in animal experiments (41, 42).

### Implications and Conclusion

An increasing number of studies suggest that OPV is associated with beneficial NSEs (7, 8, 16, 34–36). Other groups have not examined the effect of OPV campaign on child survival. We have found strong beneficial effects of OPV campaigns in several studies from urban Bissau (18, 26) and in rural areas of Guinea-Bissau and Ghana (43).

The world has experienced an unprecedented decline in child mortality in recent years. Several reviews have indicated that the decline has been much stronger in the 2000s than in the 1990s (44); for example, in Guinea-Bissau, there was no change in mortality in the 1990s but the Millenium Development Goal 4 (MDG4) of reducing child mortality by two-thirds was reached between 2000 and 2013 in the populations followed by BHP (unpublished data). Since the 2000s is the period where most campaigns have been conducted, the repeated campaigns with OPV may have been important for reaching MDG4.

This interpretation contrasts markedly with the current understanding of the decline in mortality. For example, the under-five mortality rate in Niger declined from 226/1,000 to 128/1,000 between 1998 and 2009; based on the assessment of changes in routine interventions and assumptions about their effects it was estimated that 5% of the reduction in mortality was due to improvement in routine MV and nothing to OPV or MV campaigns (45).

Since OPV in rare cases can cause vaccine-associated paralytic polio and generate circulating vaccine-derived poliovirus, it has long been the plan to stop the use of OPV. Trivalent OPV was stopped globally in April 2016. IPV is gradually being introduced to replace OPV within the next few years and bivalent and monovalent OPV should be fully phase out by 2020.

From this perspective, it is interesting that mono- and bivalent OPV appeared to have similar beneficial effects on child survival (Table 3). We have previously used IPV as a comparator vaccine in RCTs; females had significantly higher mortality than males from they received IPV and until they were given live MV (23). In a recent RCT of the effect of OPV vs IPV on diarrhea in Bangladesh, OPV was associated with less bacterial diarrheas (46). Hence, stopping OPV campaigns or replacing OPV with IPV could paradoxically lead to increases in child mortality levels. This needs to be examined before all OPV strains are stopped globally, phased out or replaced by IPV. First, it should be possible to conduct cluster-randomized trials of the overall effect on child survival of OPV-campaigns. Second, OPV0 is often not used and since we have found OPV0 to be associated with better infant survival, it should be possible to conduct RCTs of OPV0. Third, trials should compare the effect on survival of the OPV-schedule with an IPV-based schedule (34, 46). While there may be good reasons to prevent the rare side effects of OPV, there may be even better reasons to use OPV to reduce child mortality in high mortality areas.

Live vaccines may have beneficial immune training effects (3–6, 20). We need to know these effects before the target infection is eradicated and vaccinations are stopped as happened with smallpox vaccination (47, 48). Since both polio and measles infections are targeted for eradication within the next 10–20 years, there are major reasons to explore the beneficial NSEs of OPV and MV now and to examine whether similar beneficial immune-training effects can be induced in other ways.

## Ethics Statement

The study is a reanalysis of seven randomized trials conducted in Guinea-Bissau, each of them being approved by the national ethical committee and given consultative approval by the Danish national ethical committee.

## Author Contributions

AA and HR conducted the statistical analyses. AF organized data collection in connection with the campaigns. AF, AR, CM, NL, SB-S, CB, and PA supervised the individual trials and maintained the demographic surveillance system. The first draft was written by PA; all authors contributed to the final version of the paper. PA and AA will act as guarantors of the study.

## Conflict of Interest Statement

The authors declare that the research was conducted in the absence of any commercial or financial relationships that could be construed as a potential conflict of interest.

## References

[1] Higgins JPT Soares-Weiser K Reingold A Systematic Review of the Non-specific Effects of BCG, DTP and Measles Containing Vaccines. (2014). Available from: http://www.who.int/immunization/sage/meetings/2014/april/3_NSE_Epidemiology_review_Report_to_SAGE_14_Mar_FINAL.pdf

[2] Strategic Advisory Group of Experts on immunization. Week Epidemiol Rec (2014) 89:233–5. 24466571

[3] Kleinnijenhuis J Quintin J Preijers F Joosten LA Ifrim DC Saeed S Bacille Calmette-Guerin induces NOD2-dependent nonspecific protection from reinfection via epigenetic reprogramming of monocytes. Proc Natl Acad Sci U S A (2012) 109:17537–42. 10.1073/pnas.1202870109 22988082 PMC3491454

[4] Benn CS Netea MG Selin LK Aaby P A small jab – a big effect: non-specific immunomodulation by vaccines. Trends Immunol (2013) 34:431–9. 10.1016/j.it.2013.04.004 23680130

[5] Kleinnijenhuis J Quintin J Preijers F Benn CS Joosten LA Jacobs C Long-lasting effects of BCG vaccination on both heterologous Th1/Th17 responses and innate trained immunity. J Innate Immun (2014) 6:152–8. 10.1159/000355628 24192057 PMC3944069

[6] Aaby P Kollmann T Benn CS Non-specific effects of neonatal and infant vaccination – public health, immunological, and conceptual challenges. Nat Immunol (2014) 15:895–9. 10.1038/ni.2961 25232810

[7] Mogensen SW Andersen A Rodrigues A Benn CS Aaby P. The introduction of diphtheria-tetanus-pertussis and oral polio vaccine among young infants in an urban African community: a natural experiment. EBioMedicine (2017) 17:192–8. 10.1016/j.ebiom.2017.01.041 28188123 PMC5360569

[8] Aaby P Hedegaard K Sodemann M Nhante E Veirum JE Jakobsen M Childhood mortality after oral polio immunisation campaign in Guinea-Bissau. Vaccine (2005) 23:1746–51. 10.1016/j.vaccine.2004.02.054 15705481

[9] Aaby P Rodrigues A Biai S Martins C Veirum JE Benn CS Oral polio vaccination and low case fatality at the paediatric ward in Bissau, Guinea-Bissau. Vaccine (2004) 22:3014–7. 10.1016/jvaccine/2004.02.009 15297050

[10] Roth A Benn CB Ravn H Rodrigues A Lisse IM Yazdanbakhsh M Effect of revaccination with BCG in early childhood on mortality: randomised trial in Guinea-Bissau. BMJ (2010) 340:c671. 10.1136/bmj.c671 20231251 PMC2839082

[11] Benn CS Diness BR Roth A Nante E Fisker AB Lisse IM Effect of 50,000 IU vitamin A given with BCG vaccine on mortality in infants in Guinea-Bissau: randomised placebo controlled trial. BMJ (2008) 336:1416–20. 10.1136/bmj.39542.509444.AE 18558641 PMC2432170

[12] Benn CS Diness BR Balde I Rodrigues A Lausch KR Martins CL Two different doses of supplemental vitamin a did not affect mortality of normal-birth-weight neonates in Guinea-Bissau in a randomized trial. J Nutr (2014) 144:1474–9. 10.3945/jn.114.192674 24991044

[13] Aaby P Martins CL Garly ML Bale C Andersen A Rodrigues A Non-specific effects of standard measles vaccine at 4.5 and 9 months of age on childhood mortality: randomised controlled trial. BMJ (2010) 341:c6495. 10.1136/bmj.c6495 21118875 PMC2994348

[14] Aaby P Roth A Ravn H Napirna BM Rodrigues A Lisse IM Randomized trial of BCG vaccination at birth to low-birth-weight children: beneficial nonspecific effects in the neonatal period? J Infect Dis (2011) 204:245–52. 10.1093/infdis/jir240 21673035

[15] Biering-Sørensen S Aaby P Lund N Monterio I Jensen KJ Schaltz-Buchholzer F Early BCG and neonatal mortality among low-birth-weight infants: a randomised controlled trial. Clin Infect Dis (2017) 65:1183–90. 10.1093/cid/cix525 PMC5849087 29579158

[16] Lund N Andersen A Hansen AS Jepsen FS Barbosa AG Biering-Sørensen S The effect of oral polio vaccine at birth on mortality. A Randomized Trial. Clin Infect Dis (2015) 61:1504–11. 10.1093/CID/civ617 26219694 PMC4614411

[17] Fisker AB Bale C Rodrigues A Balde I Fernandes M Jørgensen MJ High-dose vitamin A with vaccination after 6 months of age: a randomized trial. Pediatrics (2014) 134(3):e739–48. 10.1542/peds.2014-0550 25136048

[18] Aaby P Andersen A Martins CL Fisker AB Rodrigues A Whittle HC Does oral polio vaccine have non-specific effects on all-cause mortality? Natural experiments within a randomised controlled trial of early measles vaccine. BMJ Open (2016) 6(12):e013335. 10.1136/bmjopen-2016-013335 28011813 PMC5223718

[19] Altman DG Andersen PK Calculating the number needed to treat for trials where the outcome is time to an event. BMJ (1999) 319:1492–5. 10.1136/bmj.319.7223.1492 10582940 PMC1117211

[20] Benn CS Fisker AB Whittle HC Aaby P. Revaccination with live attenuated vaccines confer additional beneficial nonspecific effects on overall survival: a review. EBioMedicine (2016) 10:312–7. 10.1016/j.ebiom.2016.07.016 27498365 PMC5006692

[21] Aaby P Ravn H Fisker AB Rodrigues A Benn CS. Is diphtheria-tetanus-pertussis (DTP) associated with increased female mortality? A meta-analysis testing the hypotheses of sex-differential non-specific effects of DTP vaccine. Trans R Soc Trop Med Hyg (2016) 110(10):570–81. 10.1093/trstmh/trw073 27856947 PMC5155548

[22] Garly ML Jensen H Martins CL Balé C Balde MA Lisse IM Hepatitis B vaccination associated with higher female than male mortality in Guinea-Bissau: an observational study. Pediatr Infect Dis J (2004) 23:1086–92. 15626943

[23] Aaby P Garly ML Nielsen J Ravn H Martins C Balé C Increased female-male mortality ratio associated with inactivated polio and diphtheria-tetanus-pertussis vaccines: observations from vaccination trials in Guinea-Bissau. Pediatr Infect Dis J (2007) 26:247–52. 10.1097/01.inf.0000256735.05098.01 17484223

[24] Fisker AB Biering-Sørensen S Lund N Djana A Rodrigues A Martins CL Contrasting female-male mortality ratios after routine vaccinations with pentavalent versus measles and yellow fever vaccine. A cohort study from Guinea-Bissau. Vaccine (2016) 34(38):4551–7. 10.1016/j.vaccine.2016.07.034 27475473

[25] Klein SL Shann F Moss WJ Benn CS Aaby P RTS,S malaria vaccine and increased mortality in girls. mBio (2016) 7(2):e514–6. 10.1128/mBio.00514-1 PMC4850267 27118593

[26] Benn CS Jacobsen LH Fisker AB Rodrigues A Sartono E Lund N Campaigns with oral polio vaccine may lower mortality and create unexpected results. Vaccine (2017) 35:1113–6. 10.1016/j.vaccine.2016.11.006 28139347 PMC5312669

[27] Fisker AB Rodrigues A Martins C Byberg S Thysen S Pedersen M Reduced mortality after general measles vaccination campaign in rural Guinea-Bissau. Pediatr Infect Dis J (2015) 34:1369–76. 10.1097/INF.0000000000000896 26379164

[28] Byberg S Thysen SM Rodrigues A Martins C Cabral C Careme M A general measles vaccination campaign in urban Guinea-Bissau: comparing child mortality among participants and non-participants. Vaccine (2017) 35:33–9. 10.1016/j.vaccine.2016.11.049 27890397

[29] Contreras G Sabin’s vaccine used for nonspecific prevention of infant diarrhea of viral etiology. Bull Pan Am Health Organ (1974) 8:123–32. 4369156

[30] Contreras G. Effect of the administration of oral poliovirus vaccine on infantile diarrhoea mortality. Vaccine (1989) 7:211–2. 10.1016/0264-410X(89)90230-2 2781855

[31] Voroshilova MK Potential use of nonpathogenic enteroviruses for control of human disease. In: Melnick JL, editor. Prog Med Virol. (Vol. 36), Basel: Karger (1989). p. 191–202. 2555836

[32] Lund N Biering-Sørensen S Andersen A Monteiro I Camala L Jørgensen MJ Neonatal vitamin A supplementation associated with a cluster of deaths and poor early growth in a randomised trial among low-birth-weight boys of vitamin A versus oral polio vaccine at birth. BMC Pediatr (2014) 14:214. 10.1186/1471-2431-14-214 25163399 PMC4236664

[33] Benn CS Fisker AB Rodrigues A Ravn H Sartono E Whittle H Sex-differential effect on infant mortality of oral polio vaccine administered with BCG at birth in Guinea-Bissau. A Natural Experiment. PLoS ONE (2008) 3:e4056 10.1371/journal.pone.0004056 19112511 PMC2605256

[34] Seppälä E Viskari H Hoppu S Honkanen H Huhtala H Simell O Viral interference induced by live attenuated virus vaccine (OPV) can prevent otitis media. Vaccine (2011) 29:8615–8. 10.1016/j.vaccine.2011.09.015 21939720 PMC7127548

[35] Sørup S Stensballe LG Krause TG Aaby P Benn CB Ravn H Oral polio vaccination and hospital admissions with non-polio infections in Denmark: nationwide retrospective cohort study. Open Forum Infect Dis (2016) 3:ofv204 10.1093/ofid/ofv204 26885538 PMC4751340

[36] Sørup S Benn CS Poulsen A Krause T Aaby P Ravn H. Live vaccine against measles, mumps, and rubella and the risk of hospital admissions for nontargeted infections. JAMA (2014) 311:826–35. 10.1001/jama.2014.470 24570246

[37] Welsh RM Selin LH No one is naïve: the significance of heterologus T-cell immunity. Nat Rev (2002) 2:417–26. 10.1038/nri820 12093008

[38] Mathurin KS Martens GW Kornfeld H Welsh RM. CD4 T-cell-mediated heterologous immunity between mycobacteria and poxviruses. J Virol (2009) 83:3528–39. 10.1128/JVI.02393-08 19193795 PMC2663272

[39] Alam MJ Rashid MM Kabir Y Raqib R Ahmad SM. On birth single dose live attenuated OPV and BCG vaccination induces gut cathelicidin LL37 responses at 6 week of age: a natural experiment. Vaccine (2015) 33:18–21. 10.1016/j.vaccine.2014.10.075 25444792

[40] Welaga P Oduro A Debpuur C Aaby P Ravn H Andersen A Fewer out-of-sequence vaccinations and reduction of child mortality in Northern Ghana. Vaccine (2017) 35:2496–503. 10.1016/j.vaccine.2017.03.004 28341115

[41] Huber SA Pfaeffle B. Differential Th1 and Th2 cell responses in male and female BALB/c mice infected with Coxsackievirus group B Type 3. J Virol (1994) 68:5126–32. 8035512 10.1128/jvi.68.8.5126-5132.1994 PMC236456

[42] Fischer JE Johnson JE Johnson TR Graham BS. Pertussis toxin sensitization alters the pathogenesis of subsequent respiratory syncytial virus infection. J Infect Dis (2000) 182:1029–38. 10.1086/315806 10979896

[43] Moran TM Park H Fernandez-Sesma A Schulman JL. Th2 responses to inactivated influenza virus can be converted to Th1 responses and facilitate recovery from heterosubtypic virus infection. J Infect Dis (1999) 180:579–85. 10.1086/314952 10438342

[44] Wang H Liddell CA Coates MM Mooney MD Levitz CE Schumacher AE Global, regional, and national levels of neonatal, infant, and under-5 mortality during 1990–2013: a systematic analysis of the Global Burden of Disease Study 2013. Lancet (2014) 384:957–79. 10.1016/S0140-6736(14)60497-9 24797572 PMC4165626

[45] Amouzou A Habi O Bensaïd K Niger Countdown Case Study Working Group. Reduction in child mortality in Niger: a Countdown to 2015 country case study. Lancet (2012) 380:1169–78. 10.1016/S0140-6736(12)61376-2 22999428

[46] Upfill-Brown A Taniuchi M Platts-Mills JA Kirkpatrick B Burgess SL Oberste MS Non-specific effects of oral polio vaccine on diarrheal burden and etiology among Bangladeshi children. Clin Infect Dis (2017) 65:414–9. 10.1093/cid/cix354 28444240 PMC5848225

[47] Aaby P Gustafson P Roth A Rodrigues A Fernandes M Sodemann M Vaccinia scars associated with better survival for adults. An observational study from Guinea-Bissau. Vaccine (2006) 24:5718–25. 10.1016/j.vaccine.2006.04.045 16720061

[48] Sørup S Villumsen M Ravn H Benn CS Sørensen TIA Aaby P Smallpox vaccination and all-cause infectious disease hospitalization: a Danish register-based cohort study. Int J Epidemiol (2011) 40:955–63. 10.1093/ije/dyr063 21543446

