# Supplementary material for: National Immunization Campaigns with Oral Polio Vaccine Reduce All-Cause Mortality: A Natural Experiment within Seven Randomized Trials
Source: Front Public Health. 2018 Feb 2;6:13. doi: 10.3389/fpubh.2018.00013 (PMC5801299; doi:10.3389/fpubh.2018.00013)
Supplement: Supplementary file 1 [file Data_Sheet_1.docx]

**National immunisation campaigns with oral polio vaccine reduce all-cause mortality: A natural experiment within seven randomised trials**

**Supplementary material**

The supplementary material has additional information on: I. The seven randomised trials (RCTs); II. Tables with additional information

1. **The seven randomised trials**

BHP conducted eight RCTs during the study period; one RCT (RCT 0) did not have relevant data, the other seven were used for the present analysis.

**RCT I and RCT II. Neonatal vitamin A supplementation trials, normal birth weight newborns**

Two trials of neonatal VAS versus placebo among normal-birth-weight (>2500 g) newborns were conducted between 2002 and 2007 (11,12). Children were randomised to VAS or placebo in connection with their first vaccination contact (BCG, OPV) before leaving the maternity ward or at one of the health centres in the study area. In the first trial the children received 50,000 IU vitamin A; in the second trial the children were randomised to placebo, 25,000 IU or 50,000 IU of vitamin A. The children were followed to 12 months of age. After censoring for enrolment in the early measles trials there were 150 deaths and 150 deaths in these trials.

**RCT III. Early two-dose MV trial**

The trial recruited children between 2003 and 2007 and followed the children to 3 years of age in Bandim (13). Briefly, 6417 children were randomised 1:2 to receive standard dose Edmonston-Zagreb (EZ) MV at enrolment at 4.5 months of age or no vaccine. At 9 months of age all children were invited back to receive the standard MV recommended by WHO; the two-dose group received EZ MV and the one-dose had been randomised to receive either EZ or Schwarz (SW) MV. Hence, between 4.5 and 9 months of age the trial compared the effect on mortality of one early dose of MV versus no MV (i.e. having DTP3 plus OPV3 as the most recent vaccination); between 9 and 36 months of age the trial compared two doses of MV versus one dose of MV. Only children who had received DTP3 at least 4 weeks earlier were enrolled in the early two-dose trial to prevent that DTP was given after MV. Children in the early MV trial were exempted from receiving MV in the MV campaigns. There were 217 deaths in the per-protocol analysis of this trial.

**RCT IV and RCT V. BCG at birth to low-birth children**

Low-birth-weight (LBW) children were randomised to receive BCG at birth (intervention) or delayed BCG (current policy) at the first contact with the health care system, i.e. either when leaving the maternity ward or when coming to a health centre to get vaccinated (14,15). Two trials were conducted between 2004 and 2013. In the first trial (RCT IV) infant mortality was the primary outcome (14); in RCT V neonatal mortality was the primary outcome and infant mortality a secondary outcome (15). After censoring for enrolment in the early measles trials there were 223 deaths and 320 deaths in these trials.

**RCT VI. OPV-at-birth (OPV0) trial**

The trial was conducted between 2008 and 2011. At the first contact with the health care system normal birth weight children were randomised to receive BCG+OPV0 or only BCG (16). The main outcome was infant mortality. After censoring for enrolment in the early measles trials there were 151 deaths in this trial.

**RCT VII. Trial of VAS vs placebo administered with vaccines after 6 months of age.**

Between 2007 and 2010, children in both urban and rural areas of the Bandim Health Project were randomised to receive VAS (50,000 IU < 12 months; 100,000 IU >= 12 months) versus placebo when they were due to receive routine vaccinations between 6 and 23 months of age (17). The children were followed for 12 months after enrolment. All the other RCTs were from urban areas, so only the urban part of the VAS trial has been included in the present analysis; this part of the trial only included children enrolled up to 17 months of age. There were 33 deaths in the urban part of this trial. The data from the rural areas is being included in an analysis of the effect of campaigns in the rural areas.

**RCT 0. Trial of BCG revaccination**

This trial took place from 2003-2007. However, recruitment in this trial was only at 19 months so essentially all children had received campaign OPV. There was only one death among children who had not received campaign OPV (10). The trial was therefore not included in the analysis.

**II. Tables with additional information**

**Supplementary Table 1. National child health intervention campaigns in Guinea-Bissau 2002-2014**

| **Year** | **Month** | **Interventions (Target age group)^a^** | **Coverage** |
| --- | --- | --- | --- |
| 2002 | October | Trivalent OPV (0-59 months) |  |
| 2002 | November | VAS (6-59 months) + Trivalent OPV (0-59 months) |  |
| 2003 | November | VAS and missing vaccines (6-59 months) | Children 6-17 months: 78% (A) |
| 2004 | October | Trivalent OPV (0-59 months) |  |
| 2004 | November | VAS (6-59 months) + Trivalent OPV (0-59 months) | Monitored supplementation 81% of study area population aged 6-59 months (B) |
| 2005 | November | Trivalent OPV (0-59 months) | Monitored vaccination: 80% of study area population aged 0-35 months (5821/7315)# |
| 2005 | December | VAS (6-59 months) + Trivalent OPV (0-59 months) | Monitored vaccination: 79% of study area population aged 0-35 months (5522/7012)# |
| 2006 | May | VAS (6-59 months) + MV (6 months-15 years) + Mebendazole (12-59 months) | Children 6-35 months 85% (5135/6077)* |
| 2006 | November | VAS (6-59 months) + Mebendazole (12-59 months) + ITN (0-59 months) | Children 6-35 months: Monitored supplementation:43% (2861/6653)# |
| 2007 | June-July | VAS (6-59 months) + Mebendazole (12-59 months) | Children 6-35 months 56% (2997/5331)* |
| 2007 | December | VAS (6-59 months) + Mebendazole (12-59 months) | Children 6-35 months: 58% (C) |
| 2008 | July | VAS (6-59 months) + Mebendazole (12-59 months) | Children 6-35 months: 68% (C) |
| 2009 | January | VAS (6-59 months) + Mebendazole (12-59 months) + Iodine (depending on stock) | Children 6-35 months 96% (6297/6540)* |
| 2009 | July | VAS (6-59 months) + MV (9 months-5 years) + Mebendazole (12-59 months) + missing vaccines | Children 6-35 months 92% (4241/4628) |
| 2010 | January | VAS (6-59 months) + Mebendazole (12-59 months) | Children 6-35 months Monitored supplementation:85% (5601/6552)# |
| 2010 | March | Monovalent OPV1 (0-59 months) | Children 0-59 months 95% (3218/3377) with finger marked (D) |
| 2010 | April | Monovalent OPV3 (0-59 months) | Children 0-59 months 97% (3939/4049) with finger marked (4) |
| 2010 | May-June | Monovalent OPV1 (0-59 months) + VAS (6-59 months) + Mebendazole (12-59 months) | Children 0-59 months 98% (5192/5293) with finger marked (D) |
| 2010 | October | H1N1 (6 months-5 years+pregnant women+diabetics) | Children 6-35 months 74% (3817/5180)* |
| 2010 | December | VAS (6-59 months) + Mebendazole (12-59 months) |  |
| 2011 | March | Bivalent OPV1_3 (0-59 months) | 98% (11487/11707) with finger marked (D) |
| 2011 | April-May | Trivalent OPV (0-59 months) + VAS (6-59 months) + Mebendazole (12-59 months) | 98% with finger marked (D) |
| 2011 | November | Trivalent OPV (0-59 months) + VAS (6-59 months) + Mebendazole (12-59 months) | Children 0-35 months: 95% (6136/6476)* |
| 2012 | March | Trivalent OPV (0-59 months) | Children 0-35 months: 95% (5810/6127)* |
| 2012 | July | VAS (6-59 months) + Mebendazole (12-59 months) | Children 6-35 months:95% (4558/4803)* |
| 2012 | December | VAS (6-59 months) + MV (9 months-5 years) + Mebendazole (12-59 months) | Children 6-35 months:87% (4553/5248)* |
| 2013 | May | Bivalent OPV1_3 (0-59 months) + VAS (6-59 months) + Mebendazole (12-59 months) | Children 0-35 months: 96% (5879/6147)* |
| 2013 | November | Bivalent OPV1_3 (0-59 months) + VAS (6-59 months) + Mebendazole (12-59 months) | Children 0-35 months: 96% (5626/5626)* |
| 2014 | August | VAS (6-59 months) + Mebendazole (12-59 months) | Children 6-35 months:99% (4239/4283)* |

Notes: VAS: Vitamin A Supplementation; OPV: Oral Polio Vaccine; MV: Measles vaccine; ITN: Insecticide treated bed nets.

# Participation assessed in BHP urban study area through monitoring during campaign

* Participation assessed in BHP urban study area through monitoring during campaign and home visits to assess participation status for children who were not registered to have received during the campaign.

**References**

1. Benn CS, Martins C, Rodrigues A, Ravn H, Fisker AB, Christoffersen D, Aaby P. The effect of vitamin A supplementation administered with missing vaccines during national immunization days in Guinea-Bissau. Int J Epidemiol 2009; 38:304-11.
2. Yakymenko D, Benn CS, Martins C, Diness BR, Fisker A, Rodrigues A, Aaby P. The impact of different doses of vitamin A supplementation on male and female mortality. A randomised trial from Guinea-Bissau. BMC Pediatrics 2011;11:77.
3. Fisker AB, Aaby P, Bale C, Balde I, Biering-Sørensen S, Agergaard J, Martins C, Bibby BM, Benn CS. Does the effect of vitamin A supplements depend on vaccination status? An observational study from Guinea-Bissau. BMJ Open 2012:2:e000448
4. AFRO report: http://www.polioeradication.org/Dataandmonitoring/Poliocampaignmonitoring/GuineaBissau.aspx

**Supplementary Figure 1. Goodness of fit**

The fit of the multivariable model is illustrated by graphing the average predicted mortality rates (connected lines) versus the observed mortality rates (dots) for +OPV (black) and -OPV (grey) in both the rainy and the dry season in each of the calendar years of the follow-up period. Mortality rates are deaths per 100 person-years.
